# Oral application of bulleyaconitine A attenuates morphine tolerance in neuropathic rats by inhibiting long-term potentiation at C-fiber synapses and protein kinase C gamma in spinal dorsal horn

**DOI:** 10.1177/1744806920917242

**Published:** 2020-04-14

**Authors:** Jie-Zhen Mai, Chong Liu, Zhuo Huang, Chun-Lin Mai, Xin Zhou, Jun Zhang, Xian-Guo Liu

**Affiliations:** 1 Department of Physiology and Pain Research Center, Zhongshan School of Medicine, Sun Yat-sen University, Guangzhou, China; 2 Guangdong Province Key Laboratory of Brain Function and Disease, Guangzhou, China

**Keywords:** Bulleyaconitine A, morphine tolerance, long-term potentiation, neuropathic pain, protein kinase C gamma, glial cells

## Abstract

Morphine is frequently used for the treatment of chronic pain, while long-term use of the drug leads to analgesic tolerance. At present, the prevention of the side effect remains a big challenge. Bulleyaconitine A, a diterpenoid alkaloid from *Aconitum bulleyanum* plants, has been used to treat chronic pain in China for more than 30 years. In the present study, we tested the effect of bulleyaconitine A on analgesic tolerance induced by morphine injections (10 mg/kg s.c., b.i.d.) in the lumbar 5 spinal nerve ligation model of neuropathic pain. We found that intragastrical application of bulleyaconitine A (0.4 mg/kg) 30 min before each morphine injection substantially inhibited the decrease in morphine’s inhibitory effect on mechanical allodynia and thermal hyperalgesia. Mechanistically, morphine injections further potentiated the lumbar 5 spinal nerve ligation induced long-term potentiation at C-fiber synapses in the spinal dorsal horn, a synaptic model of chronic pain. This effect was completely blocked by intragastrical bulleyaconitine A. It has been well established that activation of protein kinase C gamma and of glial cells in the spinal dorsal horn are critical for the development of opioid tolerance and neuropathic pain. We found that morphine injections exacerbated the upregulation of phospho-protein kinase C gamma (an active form of protein kinase C gamma), and the activation of microglia and astrocytes in the spinal dorsal horn induced by lumbar 5 spinal nerve ligation, and the effects were considerably prohibited by intragastrical bulleyaconitine A. Thus, spinal long-term potentiation at C-fiber synapses may underlie morphine tolerance. Oral administration of bulleyaconitine A may be a novel and simple approach for treating of opioid tolerance.

## Introduction

Morphine has been used as an analgesic for thousands of years and is still the golden standard for treating severe acute and chronic pain. However, the analgesic effect of morphine is decreased with time when repetitively administrated, a phenomenon termed analgesic tolerance.^1^ To maintain analgesia, the dose of morphine has to be increased progressively, which exacerbates its side effects, including nausea, vomiting, constipation, respiratory depression, dependence, drowsiness, pruritus, and even leads to overdose deaths.^2,3^ Currently, the treatment of morphine tolerance is still unmet in clinical practice.

It has been well established that protein kinase C (PKC) is critically involved in both morphine tolerance^4^ and chronic pain.^5^ The morphine tolerance in rats is associated with upregulation of PKCγ in the spinal dorsal horn neurons^6^ and is blocked by PKC inhibitors.^7^ The development of morphine tolerance is reduced in PKCγ mutant mice.^8^ Regarding chronic pain, it has been shown^9^ that genetic deletion of PKCγ does not affect acute pain but substantially reduce neuropathic pain, a common form of chronic pain. Activation of PKCγ interneurons in medullary dorsal horn is sufficient to induce mechanical allodynia (decreased pain threshold), a behavioral sign of neuropathic pain.^10^ Activation of PKCγ in the superficial dorsal horn is also needed for inflammatory pain.^11^ Therefore, activation of PKCγ in the spinal dorsal horn may underlie both morphine tolerance and chronic pain. Interestingly, several lines of clinical and experimental evidence show that a much higher dose of opioids is needed for treating neuropathic pain than that for treating acute (nociceptive) pain.^12,13^ The data indicate that neuropathic pain itself is a form of opioid tolerance, and neuropathic pain and opioid tolerance may share common mechanisms. Consistent with the notion, compelling evidence shows that the activation of microglia and astrocytes in the spinal dorsal horn is critically involved in both neuropathic pain and morphine tolerance.^14–16^

Bulleyaconitine A (BLA), a diterpenoid alkaloid from *Aconitum bulleyanum* plants, has been used to treat chronic pain in China, since 1985.^17,18^ Our previous studies show that BLA attenuates paclitaxel-induced neuropathic pain and depresses spinal long-term potentiation (LTP) at C-fiber synapses by inhibiting presynaptic transmitter release.^19^ BLA attenuates the mechanical allodynia and thermal hyperalgesia induced by lumbar 5-spinal nerve ligation (L5-SNL) by inhibition of tetrodotoxin-sensitive (TTX-S) voltage gate-sodium channels, especially Na_v_1.7, in dorsal root ganglion (DRG) neurons via inhibiting PKC.^20,21^ However, which isoform of PKC is affected by BLA is still unknown.

In the present study, the effect of BLA on morphine tolerance was investigated in the rats with neuropathic pain induced by L5-SNL. We found that oral administration of BLA substantially attenuated morphine tolerance by inhibiting PKCγ and glial activation in the spinal dorsal horn.

## Materials and Methods

### Animals

Male Sprague-Dawley rats (180–250 g) were housed in separate cages at a temperature-controlled (24 ± 1°C) and humidity controlled (50%–60%) room with a 12:12-h light/dark cycle. The animals had access to food and water freely and were raised in the cage with an automatic full-membrane individual ventilated caging system (IVC; XDWG-25, Suzhou Junshen Experiment Animal Equipment Ltd. Suzhou, China). All animal experimental procedures were approved by the Animal Care and Use Committee of Sun Yat-sen University and were carried out under the guideline of the National Institutes of Health on animal care and the ethical guidelines for investigation of experimental pain in conscious animals.^22^ All animals were randomly assigned to different experimental or control conditions in the current study.

### Surgical procedures

L5-SNL was conducted following the procedures described previously.^23,24^ Briefly, surgery was performed under inhalation anesthesia consisting of 1%–3% isoflurane (RWD Life Science, R510-22). The left L5 spinal nerve was isolated adjacent to the vertebral column and tightly ligated with 6–0 silk sutures distal to the DRG and proximal to the formation of the sciatic nerve. In sham operated rats, the L5 spinal nerves were identically exposed but not ligated.

### Behavioral tests and drug administration

Animals were habituated to separate transparent Plexiglas chambers positioned on a wire mesh floor for 30 min each day for consecutive three days before behavioral tests. Mechanical sensitivity was assessed before and seven days after surgery with the up–down method described previously,^25^ using a set of von Frey hairs with logarithmically incremental stiffness from 0.6–15 g (0.6, 1, 2, 4, 6, 8, 15 g). Each stimulus consisted of a 6–8 s application of the von Frey hair to the middle of the plantar surface of the foot with 5-min interval between stimuli. Quick withdrawal or licking of the paw in response to the stimulus was considered a positive response. Thermal withdrawal latency to radiant heat was determined with a previously described method^26^ using a 390 Analgesia Meter (IITC Inc., Woodland Hills, CA). Rats were placed individually into Plexiglas cubicles placed on a transparent glass surface. The light beam projects vertically from bulb, located below the glass directly at the plantar surface of each hindpaw. Hindpaw withdrawal latency was assessed as the time from the onset of radiant heat stimulation to withdrawal of the hindpaw. A cut-off time was set to 25 s to avoid additional thermal injury.

BLA powder (Yunnan Haopy Pharmaceutical Co., Ltd, Kunmin, Yunnan, China) was dissolved in 0.5% carboxymethylcellulose sodium solution to 0.1 mg/mL. Morphine hydrochloride (Qing-hai Pharmaceutical Factory, Xining, PR China) was dissolved in 0.9% saline at 10 mg/mL. To determine the effect of BLA on morphine tolerance, morphine (10 mg/kg) was subcutaneously injected twice a day for 10 days, according to previous studies,^27,28^ and BLA was administrated intragastrically (0.4 mg/kg) 30 min before each morphine injection.

### Recording of C-fiber-evoked field potentials

C-fiber-evoked field potentials in the spinal dorsal horn were recorded as described previously.^29^ In brief, under anesthesia with urethane (1.5 g/kg, i.p.), a laminectomy was performed to expose the lumbar enlargement of the spinal cord, and the left sciatic nerve was dissected free for electrical stimulation with a bipolar platinum hook electrode. The rats were placed in a stereotaxic frame for electrophysiological recording. The field potentials were recorded at a depth of 100–400 μm from the surface of the spinal cord in ipsilateral lumbar enlargement (L4 and L6 segments) with a glass microelectrode, which was driven by an electronically controlled microstepping motor (Narishige Scientific Instrument Laboratory). An A/D converter card (ADC-42. PICO) was used to digitize and store data at a sampling rate of 10 kHz. The strength of the test stimulation (0.5 ms duration, every 1 min) was adjusted to 1.5–2 times of threshold for C-fiber response. The amplitudes of C-fiber evoked field potentials were determined on-line by the LTP program (www.ltp-program.com).

### Western blot

The spinal dorsal horn was harvested under 0.4% sodium pentobarbital anesthesia (40 mg/kg body weight, i.p.). The tissues were homogenized and ultrasound on ice in sodium dodecyl sulfate (SDS) lysis buffer (Beyotime P00013C) with protease inhibitor cocktail (Roche Molecular Biochemicals) and phosphatase inhibitor (4906837001, Roche Molecular Biochemicals), followed by centrifugation at 14,800 r/min for 30 min at 4°C. Total protein concentration was determined by Bicinchoninic acid protein assay (Pierce, Rockford, IL, USA).

The protein samples were separated via gel electrophoresis (SDS-PAGE) and transferred onto a PVDF membrane. After blocking at room temperature for 1 h, membranes were incubated with primary antibody *p*-PKC γ (1:500, Abcam) overnight at 4°C. Followed by washing three times and incubation with horseradish peroxidase-conjugated IgG (Cell Signaling Technology). Immunolabeling was detected by enhanced chemiluminescence (Bio-Rad) and imaged using a Tanon-5200 Chemiluminescent Imaging System (Tanon Science and Technology). The band was quantified with a computer-assisted imaging analysis system (ImageJ; National Institutes of Health, USA)

### Immunofluorescent staining

Rats were deeply anesthetized with 0.4% sodium pentobarbital anesthesia (40 mg/kg body weight, i.p.) and perfused transcardially with 500 ml of cold phosphate-buffered saline (PBS) followed by 500 ml of cold 4% paraformaldehyde (PFA). The spinal cord was removed and postfixed with the same 4% PFA for 1–2 h at 4°C and then transferred to 30% sucrose in PBS overnight. Sample sections (20 μm thickness) were adhered on gelatin-coated glass slide with a cryostat (Leica). The sections were washed three times with PBS pH = 7.35–7.40 and then hatched in PBST (0.3% Triton in PBS) for 40 min, blocked with QuickBlock buffer (Beyotime, P0260) for 10 min, subsequently incubated overnight at 4°C with primary antibody for rabbit-anti-*p*-PKC (1:200, Abcam), mouse-anti-CGRP (1:200, Abcam), goat-anti-GFAP (1:200, Abcam), mouse-anti-OX-42(1:200, Abcam), anti-IB4(1:50, Sigma), and mouse-anti-NeuN (1:200, Millipore). The sections were then incubated for 60 min at room temperature with secondary antibodies Cy3-conjugated donkey anti-rabbit IgG (1:200, Jackson ImmunoResearch), FITC-conjugated donkey anti-goat IgG (1:200, Jackson ImmunoResearch), and Alexa 488-conjugated donkey anti-mouse IgG (1:200, Thermofisher). The stained sections were captured with LSM 780 (Carl Zeiss). The fluorescent density was quantified with a computer-assisted imaging analysis system (ImageJ, National Institutes of Health).

### Statistical analysis

All data were presented as means ± SEM and means ±SD. The data of the behavioral tests and field potential recordings between groups were compared using two-way analysis of variance (ANOVA) followed by a Tukey post hoc test. The relative densities of Western blots and immunofluorescence were analyzed via one-way ANOVA accompanied by a Tukey post hoc test among groups. Statistical analysis was performed with SPSS 23.0.

## Results

### Oral application of BLA attenuates morphine tolerance in the rats with Lumbar 5 spinal nerve ligation

**Figure 1.**
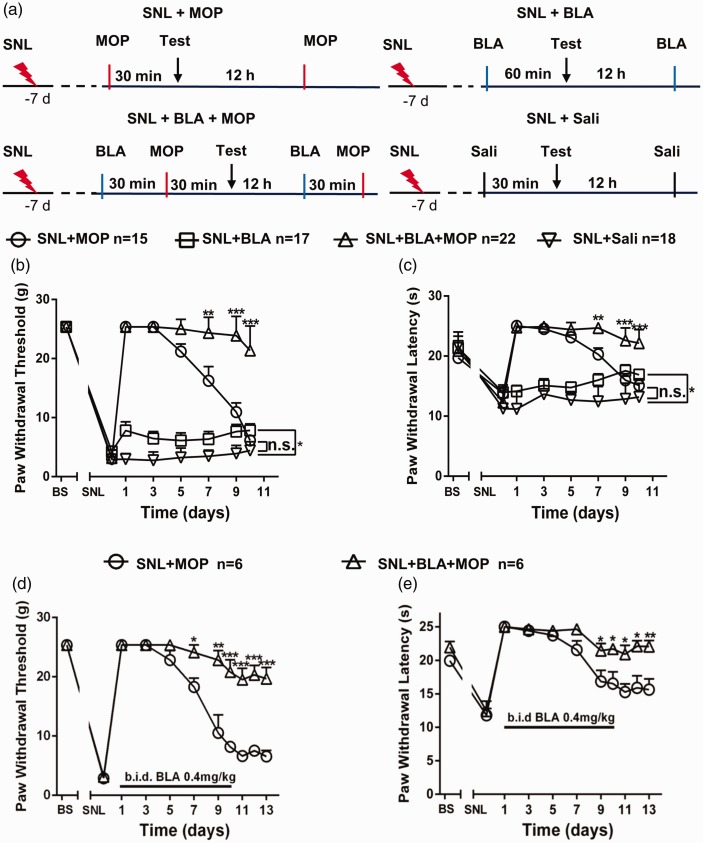
Oral application of BLA prevents morphine tolerance in the rats with L5-SNL. (a) Experimental schedules are shown. (b) and (c) The changes in paw withdrawal thresholds and paw withdrawal latencies at different time points in indicated groups are shown. Sali: saline. (d) and (e) The data show that morphine was still highly effective for inhibition of mechanical allodynia and thermal hyperalgesia after termination of BLA administration on day 10. *P <* *0.05, **P <* *0.01, ***P <* *0.001, compared with SNL + BLA + MOP group. Two-way variance (ANOVA) followed by a Tukey post hoc test.

To test the effects of intragastrical BLA on the morphine tolerance in neuropathic rats, seven days after L5-SNL surgery when mechanical allodynia (decrease in paw withdrawal threshold, PWT) and thermal hyperalgesia (decrease in paw withdrawal latency, PWL) were fully established (Figure 1(b) to (e)), animals were randomly divided into the following four groups. SNL + morphine (MOP): receiving morphine (10 mg/kg, s.c., b.i.d. for 10 days), according to previous studies;^27,28^ SNL + BLA: receiving oral BLA (0.4 mg/kg); SNL + BLA + MOP: receiving morphine injection 30 min after BLA; SNL + saline: receiving only saline injection. The behavioral tests were performed on day 1, 3, 5, 7, 9, and 10 at 30 min after morphine or saline injection or 60 min after oral BLA (see Figure 1(a) for details). As shown in Figure 1(b) and (c), in SNL + MOP group, the mechanical allodynia (decreased PWTs) and the thermal hyperalgesia (decreased PWLs) were reversed on day 1 and day 3, and then the analgesic effects reduced gradually. On day 10, the morphine analgesic effect completely disappeared, as PWTs and PWLs were no longer different from those in SNL + saline control group. In SNL + BLA + MOP group, however, the analgesic effect remained at a high level throughout the experiments. The data indicate that morphine analgesic tolerance is developed with time, and oral BLA substantially attenuates the morphine tolerance. In SNL + BLA group, only a small but significant inhibitory effect on PWTs and PWLs was observed, indicating that persist analgesic effect in SNL + BLA + MOP group might result from inhibition of morphine tolerance but not from analgesic effect of BLA. To confirm this, in another cohort of rats, oral BLA was discontinued on day 10, and morphine was injected for three days. In the absence of BLA, the effect of morphine was still persisted. While in SNL + MOP group, morphine remained ineffective during this period time (Figure 1(d) and (e)).

### Morphine further potentiates the spinal LTP induced by L5-SNL, and the effect is blocked by oral administration of BLA

**Figure 2.**
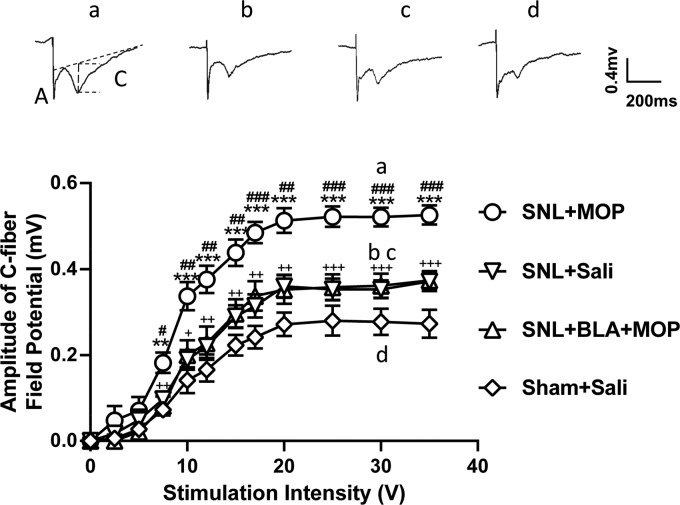
Oral application of BLA prevents the further potentiation of spinal LTP at C-fiber synapses induced by morphine in L5-SNL rats. The stimulus–response curves of C-fiber–evoked field potentials in different groups as indicated are shown (*n* = 6 per group). The raw traces show the representative recordings of the field potentials evoked by 30 V (0.5 ms in duration) in different groups as indicated. (a) and (c) indicate A-fiber and C-fiber responses, respectively. The vertical dot line shows the amplitude of C-fiber evoked field potential detected by LTP program. *P <* *0.05, **P <* *0.01, ***P <* *0.001, compared with the SNL + Saline group; ^#^*P *<* *0.05, ^##^*P *<* *0.01, ^###^*P *<* *0.001, compared with the SNL + BLA + MOP groups; +*P *<* *0.05, ++*P *<* *0.01, +++*P *<* *0.001, compared with the Sham + Saline group. Two-way analysis of variance (ANOVA) followed by a Tukey post hoc test.

Previous studies show that LTP at C-fiber synapses in the spinal dorsal horn may underlie chronic pain^30,31^ and opioid-induced hyperalgesia.^32^ To determine if BLA may prevent morphine tolerance by blocking the spinal LTP, C-fiber field potentials evoked by electrical stimulation of the sciatic nerve at different intensities were recorded 10–15 days after administration of drugs and saline, and stimulus-response curves in different groups were calculated. The results showed that the curve in SNL + saline group was significantly shifted leftward compared to sham-operated rats treated with saline (Figure 2, inverse triangles vs. diamonds), indicating that L5-SNL induces the spinal LTP at C-fiber synapses. We found that morphine further potentiated spinal LTP in L5-SNL rats, as the leftward shift of the stimulus-response curve was more robust in SNL + MOP group, compared to MOP + saline group (Figure 2, inverse triangles vs. circles). In SNL + BLA + MOP group (triangles), however, the leftward shift was significantly smaller than that in SNL + MOP group and was not different from that in SNL + saline group. The data indicate that the further potentiation induced by morphine in L5-SNL rats is completely blocked by BLA.

### L5-SNL and morphine injections upregulate *p*-PKCγ in the spinal dorsal horn neurons, which is blocked by oral administration of BLA

**Figure 3.**
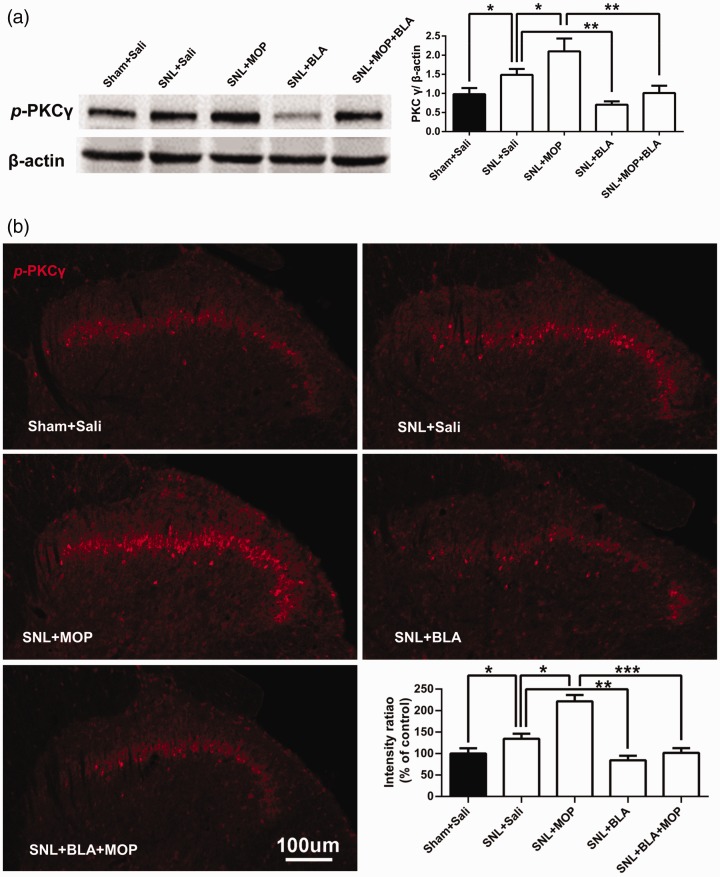
Oral application of BLA inhibits upregulation of *p*-PKCγ in spinal dorsal horn induced by both L5-SNL and morphine. (a) The Western blots were performed using the ipsilateral spinal dorsal horn tissues from the different groups following the behavioral tests shown in Figure 1. The histograms show quantification of *p*-PKCγ in different groups. *n* = 5–6 rats per group. (b) The confocal microscopy images show that *p*-PKCγ is expressed mainly in laminae II of spinal dorsal horn. Scale bars: 100 μm. The histograms show the statistic comparisons of fluorescence densities in different groups as indicated. *n* = 5 rats in each group, three images per animal. *P <* *0.05, **P <* *0.01, ***P <* *0.001. One-way variance (ANOVA) followed by a Tukey post hoc test.

**Figure 4.**
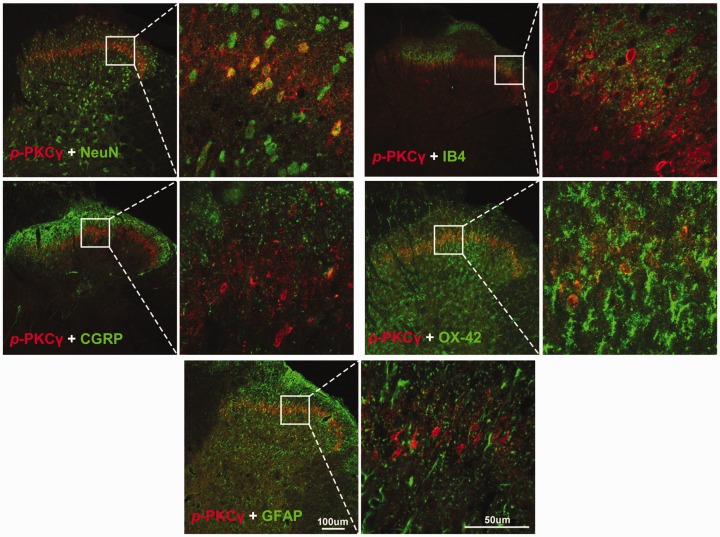
*p*-PKCγ is only expressed in spinal dorsal horn neurons. The confocal microscopy images of double staining show that *p*-PKCγ was colocalized with NeuN, but not with CGRP, IB4, OX-42, and GFAP.

Previous studies show that PKCγ is essential for development of the neuropathic pain^9^ and of morphine tolerance.^6,8^ We, therefore, investigated whether BLA may attenuate morphine tolerance by inhibiting the activation of PKCγ produced by L5-SNL and morphine. To this end, the levels of *p*-PKCγ, an active form of PKCγ, in ipsilateral spinal dorsal horn of different groups were assessed following the behavioral tests (shown in Figure 1). Western blots revealed that the level of *p*-PKCγ was higher in SNL + saline group compared to Sham + saline group, indicating that L5-SNL activates PKCγ in dorsal horn (Figure 3(a)). The PKCγ activity was potently depressed by BLA, as *p*-PKCγ level in SNL + BLA group was lower compared not only with that in SNL + saline group but also with Sham + saline group. Similar to the spinal LTP, morphine also further enhanced PKCγ activation induced by L5-SNL, as *p*-PKCγ level was higher in SNL + MOP group than that in SNL + saline group. The PKCγ activation induced by combination of L5-SNL and morphine was completely blocked by BLA, as the *p*-PKCγ level in SNL + BLA + MOP group was lower compared with that SNL + MOP group, and was not different from that in Sham + saline group (Figure 3(a)). In consistence with a previous study,^8^ immunostaining showed that *p*-PKCγ was expressed in lamina II of dorsal horn. Analysis of *p*-PKCγ fluorescence densities in the different groups revealed the similar results as Western blots, i.e., BLA blocked the PKCγ activation induced by both L5-SNL and morphine (Figure 3(b)). Double staining showed that *p*-PKCγ was only colocalized with NeuN (a marker for neuron), but not with CGRP (a marker for peptidergic afferent C fiber), IB4 (a marker for non-peptidergic afferent C fiber), OX-42 (a marker for microglia), and GFAP (a marker for astrocyte; Figure 4).

### Oral application of BLA inhibits activation of microglia and astrocytes induced by L5-SNL and morphine

**Figure 5.**
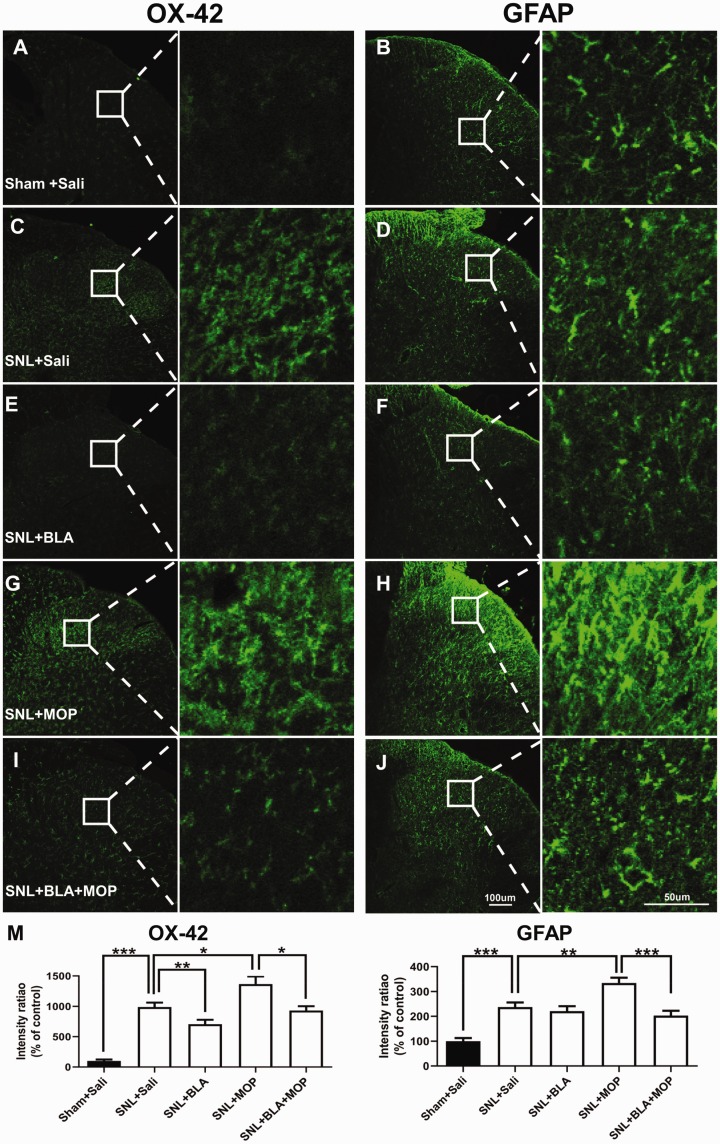
Oral application of BLA depresses the activation of glial cells in the spinal dorsal horn induced by L5 SNL and morphine. (a)–(j): Confocal images show OX-42 and GFAP expression in the ipsilateral spinal dorsal horn in different groups as indicated. (m) Quantification of OX-42 and GFAP immunofluorescence in different groups are shown. *n* = 5 rats in each group, three images per animal. *P <* *0.05, **P <* *0.01, ***P <* *0.001, one-way analysis of variance (ANOVA) followed by a Tukey post hoc test.

Previous studies show that activation of microglia^14^ and astrocytes^15^ in spinal dorsal horn is required in both the neuropathic pain and morphine tolerance. To further investigate the cellular mechanisms, by which BLA may attenuate morphine tolerance in L5-SNL rats, the glial activation in spinal dorsal horn was examined in the different groups following behavioral tests. The fluorescent densities of OX-42 (a marker for microglia) and GFAP (a marker for astrocytes) were significantly higher in SNL + saline group, compared with Sham +saline group (Figure 5(a) to (d) and (m)). The data are in line with previous studies that peripheral injury activates microglia and astrocytes in the spinal dorsal horn.^16,33,34^ Both microglia and astrocytes exhibited a morphological switch from “resting” forms with extensive thin ramifications to moderate hypertrophic shapes in L5-SNL rats. We found that the glial activation induced by L5-SNL was substantially depressed by oral application of BLA (Figure 5(c) to (f) and (m)). Again, we found morphine enhanced the effect of morphine, i.e. exaggerating the glial activation induced by L5-SNL, as the fluorescent densities of OX-42 and GFAP, were higher in SNL + MOP group (Figure 5(g), (h), and (m)) than those in SNL + saline group (Figure 5(c), (d), and (m)). The glial activation induced by combination of L5-SNL and morphine was again significantly depressed by BLA (Figure 5(g) to (j) and (m)). Therefore, inhibition of spinal glial activation may also contribute to BLA’s effect on morphine tolerance.

## Discussion

In the present work, we showed that intragastrical administration of BLA 30 min before morphine injections substantially attenuated morphine tolerance in the rats with neuropathic pain induced by L5-SNL (Figure 1). The morphine injections further potentiated LTP at C-fiber synapses in spinal dorsal horn induced by L5-SNL, and the effect was completely blocked by oral application of BLA (Figure 2). Repetitive application of morphine aggravated the activation of PKCγ and of microglia and astrocytes in the dorsal horn induced by L5-SNL, the effects were substantially depressed by BLA (Figures 3 and 5). Together, the spinal LTP at C-fiber synapses may underlie the morphine tolerance. BLA may inhibit morphine tolerance by depressing activation of PKCγ and of glial cells in spinal dorsal horn.

In this present study, the effect of BLA on morphine tolerance was investigated in the neuropathic rats but not in naïve ones, because repetitive dosing of morphine is commonly used in chronic pain patients.

### Roles of LTP at C-fiber synapses in the spinal dorsal horn in chronic pain and in the morphine tolerance

C-fiber, also called pain fiber, conducts nociceptive signals from peripheral nociceptors to spinal dorsal horn. The spinal LTP at C-fiber synapses, discovered in 1995,^29^ can be induced only by the noxious events, such as intensive electrical stimulation sufficient to activate afferent C-fibers,^35^ peripheral nerve injury,^36^ and tissue inflammation.^37^ The spinal LTP and chronic pain share almost identical cellular and molecular mechanisms.^38,39^ Activation of microglia and astrocytes is needed for induction of the spinal LTP.^40–42^ Importantly, LTP-inducible conditioning stimulation produces a long-lasting behavioral signs of pathological pain in human subjects^43^ and in rodents.^30^ Thus, the spinal LTP is proposed to underlie chronic pain.^31^

Compelling clinical and experimental evidence shows that single dosing of opioids induces hyperalgesia,^44,45^ and continuous use of opioid induces analgesic tolerance.^1^ The mechanisms underlying the paradoxical effect are still not fully understood. It has been shown that single dosing of morphine is sufficient to induce the spinal LTP at C-fiber synapses.^32,46^

Conventionally, to measure LTP, the efficiency of synaptic transmission is compared before and after conditioning stimulation in the same animal. As the recording time in anesthetized animals is limited (around 10 h), this method cannot tell how long LTP persists. Our previous works show that the stimulation-response curve of C-fiber evoked field potentials is reliably shifted leftwards after LTP induction.^30,35^ This allows us to compare the efficiency of C-fiber mediated synaptic transmission at any time points after LTP induction among different groups of animals. In the present work, the stimulation-response curves of C-fiber evoked field potentials in different groups were calculated 10 days after drugs and saline application (Figure 2). We found that the curve in SNL + saline group shifted leftwards compared with Sham + saline group, and the shift was more robust in SNL + MOP group than that in SNL + saline group. Thus, L5-SNL induces a persistent LTP that lasts for at least 10 days, and the spinal LTP is enhanced by morphine. Furthermore, it has been shown that the inhibitory effect of morphine on C-fiber evoked field potential is substantially reduced after LTP induction, and a much higher dose is needed for achieving the same effect as in naïve animals.^47^ Therefore, the spinal LTP at C-fiber synapses may also serve as a synaptic model of opioid-induced hyperalgesia and opioid tolerance.

### BLA inhibits morphine tolerance by depressing PKCγ and glial activation in the spinal dorsal horn

As mentioned in Introduction section, activation of PKCγ is critically involved in both neuropathic pain^9^ and morphine tolerance.^7^ Our previous work shows that oral BLA at a dose of 0.4 mg/kg inhibits thermal hyperalgesia but not mechanical allodynia induced by paclitaxel, while at a dose of 0.8 mg/kg inhibits both of them, when tested 2 h after BLA application.^19^ In the present work, we showed that intragastrical BLA at 0.4 mg/kg, which attenuated both mechanical allodynia and hyperalgesia as tested 1 h after administration, completely blocked the upregulation of *p*-PKCγ in spinal dorsal horn induced by L5-SNL and morphine. BLA also substantially depressed the activation of spinal microglia and astrocytes, which plays important roles in both the neuropathic pain and morphine tolerance.^14–16^ Thus, BLA may prevent morphine tolerance by depressing PKCγ and glial cells in the spinal dorsal horn.

How can activation of PKCγ and glial cells in the spinal dorsal horn produce morphine tolerance? Our previous studies show that microglial activation is indispensable for LTP at C-fiber synapses.^30,38,42^ Activation of PKC is also required for induction of the spinal LTP.^48^ It has been shown that repetitive application of morphine fails to activate spinal astrocytes in mice lacking the PKCγ gene.^49^ Therefore, morphine, a potent analgesic, may induce the spinal LTP at C-fiber synapses by activation of PKCγ that activates glial cells. A previous work shows that in spinal dorsal horn, PKCγ is expressed only in interneurons but not in peptidergic (CGRP) and non-peptidergic (IB4) afferent C fibers.^50^ Consistently, the present work revealed that *p*-PKCγ was exclusively expressed in the spinal interneurons but not in afferent C-fibers, microglia, and astrocytes. Accordingly, we speculated that the PKCγ-expressing interneurons might release some substances that activate glial cells in spinal dorsal horn. Further studies are needed to clarify this issue.

The mechanisms, by which BLA may depress PKCγ in the spinal dorsal horn is unclear at present. Previous studies show that BLA inhibits neuropathic pain by blocking TTX-S sodium channels, especially Na_v_1.7 and Na_v_1.3,^20,21,51,52^ which are predominantly expressed in primary afferent neurons and are upregulated in chronic pain conditions.^53–56^ The primary afferent fibers, especially A-fibers, discharge spontaneously following peripheral nerve injury.^24,57^ The ectopic discharges mediated by abnormal expression of sodium channels^58^ are critical for development of neuropathic pain.^59^ Interestingly, it has been shown that the spinal PKCγ expressing interneurons is activated only by innocuous inputs that are conducted by A-fibers.^50^ Accordingly, we proposed that blocking the Na_v_1.7 and Na_v_1.3 in afferent neurons including A-fibers might contribute to the inhibitory effect of BLA on PKCγ. Further studies are needed to elucidate this issue.

In conclusion, BLA, a monomeric compound that has been used for treating chronic pain, inhibits morphine tolerance by depressing PKCγ and glial cells in spinal dorsal horn.
